# Lateral heterostructures of WS_2_ and MoS_2_ monolayers for photo-synaptic transistor

**DOI:** 10.1038/s41598-024-57642-6

**Published:** 2024-03-22

**Authors:** Jaeseo Park, Jun Oh Kim, Sang-Woo Kang

**Affiliations:** 1 Strategic Technology Research Institute, Korea Research Institute of Standards and Science, Daejeon, 34113 Republic of Korea; 2 Precision Measurement, University of Science and Technology, Daejeon, 34113 Republic of Korea

**Keywords:** Lateral heterostructures, MoS_2_, WS_2_, Photo-transistors, Synaptic devices, Electronic properties and materials, Two-dimensional materials

## Abstract

Von Neumann architecture-based computing, while widely successful in personal computers and embedded systems, faces inherent challenges including the von Neumann bottleneck, particularly amidst the ongoing surge of data-intensive tasks. Neuromorphic computing, designed to integrate arithmetic, logic, and memory operations, has emerged as a promising solution for improving energy efficiency and performance. This approach requires the construction of an artificial synaptic device that can simultaneously perform signal processing, learning, and memory operations. We present a photo-synaptic device with 32 analog multi-states by exploiting field-effect transistors based on the lateral heterostructures of two-dimensional (2D) WS_2_ and MoS_2_ monolayers, formed through a two-step metal–organic chemical vapor deposition process. These lateral heterostructures offer high photoresponsivity and enhanced efficiency of charge trapping at the interface between the heterostructures and SiO_2_ due to the presence of the WS_2_ monolayer with large trap densities. As a result, it enables the photo-synaptic transistor to implement synaptic behaviors of long-term plasticity and high recognition accuracy. To confirm the feasibility of the photo-synapse, we investigated its synaptic characteristics under optical and electrical stimuli, including the retention of excitatory post-synaptic currents, potentiation, habituation, nonlinearity factor, and paired-pulse facilitation. Our findings suggest the potential of versatile 2D material-synapse with a high density of device integration.

## Introduction

As the third wave of artificial intelligence unfolds amidst the burgeoning volume of data and information to be processed, the pursuit of increased processing speed and a higher density of device integration has become critical requirements for information and communication technologies. The von Neumann architecture, a conventional computer architecture that segregates arithmetic/logic and memory operations, has been widely successful in personal computers and embedded systems. Its adoption has significantly contributed to the evolution of modern computing paradigms. However, this architecture often encounters significant challenges such as the von Neumann bottleneck (i.e., delay phenomenon) and limitations in enhancing the degree of device integration^1^.

In contrast, the neuromorphic computing architecture, inspired by biological neural networks in the human brain and designed to seamlessly integrate arithmetic, logic, and memory operations, is considered a promising solution. This offers cost-effective and superior performance with advantages such as reduced power consumption, compact size, and high-speed processing^2,3^. An artificial synaptic device serves as a basic building block of the neuromorphic system, emulating the essential functions of a biological synapse. Initially, non-volatile memories such as resistive random access memory^4,5^, phase change memory^6^, and ferroelectric random access memory^7,8^ have been extensively studied as representative synaptic devices based on the two-terminal structure. However, two-terminal synaptic devices are limited in their ability to emulate biological synaptic functions because their structural limitations prevent them from simultaneously performing signal transmission and learning operations^9–11^. Furthermore, they present substantial reliability challenges, especially in terms of retention and endurance.

On the other hand, three-terminal synaptic transistors, consisting of gate, source, and drain electrodes, have been developed to provide a higher degree of freedom in emulating synaptic behaviors. This advancement is achieved by leveraging an additional gate terminal, analogous to neurotransmitters, during the signal transmission process from a source terminal (i.e., pre-synaptic neuron) to a drain terminal (i.e., post-synaptic neuron)^12–15^. Significant efforts have also been dedicated to investigating opto-electronic synaptic transistors as a means to implement artificial synapses that offer high speed, wide bandwidth, low power consumption, using input light stimuli^13–17^.

Recently, advanced synaptic approaches have utilized the unique intrinsic physical properties of two-dimensional (2D) materials such as graphene, hexagonal boron nitride (h-BN), black phosphorus, and transition metal dichalcogenides (TMDCs)^18–28^. These materials have garnered considerable attention due to their atomically thin layered structures, high carrier mobility, tunable electronic and optical properties, and charge storage capabilities, offering potential advantages in device miniaturization and integration^18–31^. In particular, TMDCs are employed in photo-synaptic transistors leveraging their photoresponsivity due to their strong light-matter interactions. However, the achievement of long-term plasticity in the synaptic functions faces challenges due to low trap densities and weak charge trapping^18–25,32–34^. Therefore, refining the design and fabrication process, such as the insertion of the charge storage layer (e.g., h-BN, perylene-3,4,9,10-tetracarboxylic dianhydride, WO_3_) and substitution of the charge trapping layer (e.g., SiN_x_), has been proposed to more accurately mimic the potentiation and depression behaviors of synaptic connections by controlling charge movement within these layers^20–25,32–34^.

In this study, we present a photo-synaptic device with analog multi-states of long-term plasticity and high recognition accuracy, derived from a 2D TMDC heteronanostructure-based field-effect transistor (FET). The approach involves exploiting the lateral heterostructures of 2D WS_2_ and MoS_2_ monolayer as a photoactive channel, which offers inherently high photoresponsivity while still maintaining reliable electrical properties^29,30^. In the lateral heterojunction, the presence of the WS_2_ monolayer with large trap densities results in enhanced efficiency of charge trapping at the interface between the WS_2_/MoS_2_ heterostructures and SiO_2_ dielectric layer^31^. This enables the laterally-heterostructured WS_2_/MoS_2_-based photo-synaptic transistor (hereafter referred to as LHWM-synapse) to emulate the photo-synaptic functions. Under optical and electrical stimuli, synaptic characteristics, including retention of excitatory post-synaptic currents, potentiation, habituation, nonlinearity factor, and paired-pulse facilitation, were investigated to support the feasibility of LHWM-synapse. This advanced approach can extend their application potential across diverse fields, including neuromorphic computing, optoelectronic computing, intelligent sensing, human–machine interfaces, biomedical diagnostics, and wearables, enabling a new era in technology integration and applications^35–37^.

## Results and discussion

**Figure 1:**
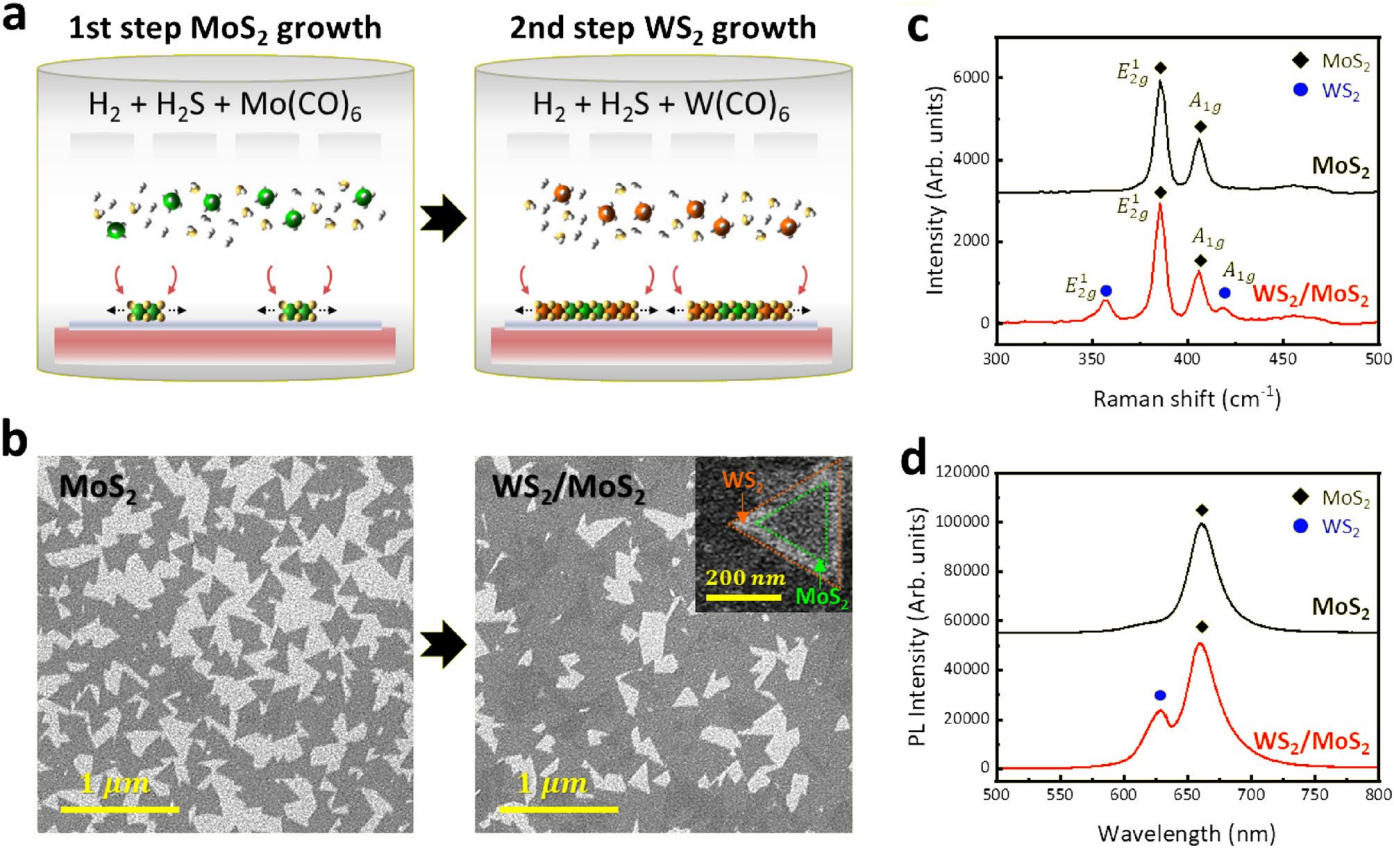
Growth and characterization of MoS_2_ and laterally-heterostructured WS_2_/MoS_2_ nanoflakes. (**a**) Schematic illustration of the growth of the WS_2_/MoS_2_ nanoflakes through a two-step sequential growth process. (**b**,**c**) Structural and optical properties of MoS_2_ and WS_2_/MoS_2_ nanoflakes: (**b**) SEM images with a magnified view in the inset, (**c**) Raman spectra, and (**d**) PL spectra.

To investigate the structural and optical properties of a laterally-heterostructured WS_2_/MoS_2_ monolayer, we characterized it using the MOCVD system (Fig. 1). The lateral heterostructures were grown using a two-step MOCVD process in a simple sequential way (Fig. 1a and Experimental Section). First, the MoS_2_ monolayer was grown on a SiO_2_/Si substrate using H_2_ ambient gas and excessive H_2_S gas relative to the Mo(CO)_6_ precursor^38–43^. Then, the injection of the Mo(CO)_6_ precursor was stopped while the flow of H_2_S and H_2_ gases was maintained under a working pressure of 40 Torr. After stabilization to purge the precursor and byproducts, the laterally-heterostructured WS_2_/MoS_2_ monolayer was grown as a monolayered WS_2_ formed at the unpassivated edges of the MoS_2_ monolayer by injecting the W(CO)_6_ precursor in the second step^38,43,44^. Top-view SEM images show that the MoS_2_ monolayers and heterostructured WS_2_/MoS_2_ monolayers are formed with sharp triangular-shaped islands (Fig. 1b). The WS_2_/MoS_2_ exhibits a dark triangular center for MoS_2_ and a bright outer edge for WS_2_. This contrast is attributed to the higher atomic number of W in WS_2_ compared to Mo in MoS_2_.

The heterostructure formation and optical properties were confirmed by Raman and photoluminescence (PL) analyses. From the Raman spectra (Fig. 1c), single-structured MoS_2_ monolayers showed two major Raman modes with the in-plane vibration ($${E}_{2g}^{1}$$) at 386.33 cm^−1^ and out-of-plane $$({A}_{1g})$$ at 405.95 cm^−1^ vibrations of S–Mo–S for MoS_2_ (i.e., black rhombus). The heterostructured WS_2_/MoS_2_ monolayers showed two major Raman modes with the $${E}_{2g}^{1}$$ at 355.35 cm^−1^ and $${A}_{1g}$$ at 417.24 cm^−1^ vibrations of S-W-S for WS_2_ (i.e., blue circle) in addition to Raman modes for the MoS_2_ monolayer. The frequency difference ($$\Delta k= {A}_{1g}- {E}_{2g}^{1}$$) between the two types of Raman vibration modes and the intensity ratio of the two peaks $$({I}_{{E}_{2g}^{1}}$$*/*$${I}_{{A}_{1g}})$$ were examined to estimate the number of layer for MoS_2_ and WS_2_^45–47^. As a result, the low $$\Delta k$$ of 19.62 cm^−1^ and 61.89 cm^−1^ and high $${I}_{{E}_{2g}^{1}}$$*/*$${I}_{{A}_{1g}}$$ of 2.38 and 1.46 for MoS_2_ and WS_2_ support the formation of lateral heterostructures of MoS_2_ and WS_2_ monolayers. Furthermore, the laterally-heterostructured WS_2_/MoS_2_ monolayer indicates the full width at half maximum (FWHM) values of 6.32 ($${E}_{2g}^{1}$$) and 6.85 ($${A}_{1g}$$) for MoS_2_ and 7.25 ($${E}_{2g}^{1}$$) and 8.14 ($${A}_{1g}$$) for WS_2_, corresponding to high crystallinity^48^.

From the PL spectra (Fig. 1d), single-structured MoS_2_ showed one PL peak observed at 661 nm (1.88 eV) for MoS_2_ (i.e., black rhombus). The laterally-heterostructured WS_2_/MoS_2_ show two different PL peaks at 628 nm (1.97 eV) and 661 nm (1.88 eV) for WS_2_ (i.e., blue circle) and MoS_2_, respectively, resulting from a direct excitonic transition by the semiconducting behavior in the WS_2_ and MoS_2_ monolayers with 2H-phase^49,50^. Since there is no PL quenching caused by the formation of the vertically heterostructure in the spectrum of WS_2_/MoS_2_ heterostructure, these results support that the lateral heterostructures of WS_2_/MoS_2_ monolayer are well-formed by a two-step process of simple sequential MOCVD growth.

**Figure 2:**
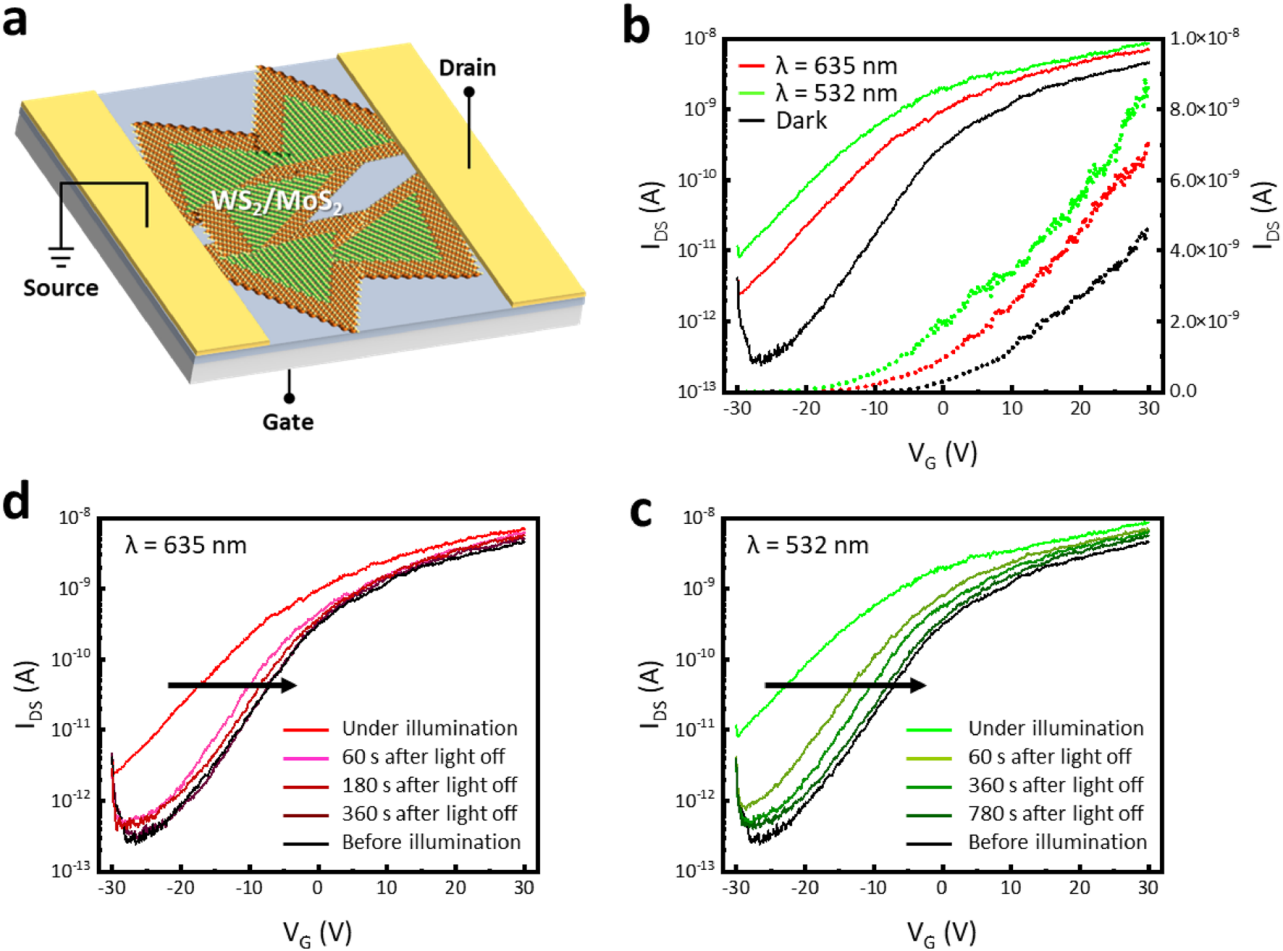
Device structure and transfer characteristics of the WS_2_/MoS_2_-based back-gated FET. (**a**) Schematic illustration of back-gated WS_2_/MoS_2_-based photo-transistor. Transfer characteristics with a wavelength of (**b**) 635 nm and (**c**) 532 nm.

To examine the feasibility of the lateral heterostructures of WS_2_/MoS_2_ monolayer as a photoactive channel, we fabricated a back-gated FET using a conventional lithography and metal deposition process (Experimental Section). The electrical properties of the laterally-heterostructured WS_2_/MoS_2_-based FET (hereafter referred to as LHWM-FET) were investigated for a photo-synapse (Fig. 2). In the dark condition, the output characteristic of the LHWM-FET exhibits the Ohmic and n-type behaviors between WS_2_/MoS_2_ channel and Au/Ti electrodes (Figure S1a). In the dark and light illumination, the transfer characteristics were explored in a logarithmic scale, where the drain-source current (I_DS_) is plotted as a function of the gate voltage (V_G_) at the source-drain voltage (V_DS_) of + 1 V (Fig. 2b).

The I_DS_-V_G_ transfer characteristic in dark condition shows that the LHWM-FET has n-type and clockwise hysteresis behaviors that originated from the carrier traps (Figure S1b). From the I_DS_–V_G_ transfer characteristic of the LHWM-FET, the ratio of maximum I_DS_ to minimum I_DS_ (on/off ratio) of ~ 10^4^, threshold voltage (V_TH_) of − 19.45 V, and sub-threshold swing (SS) of 10.25 V/dec were calculated (Experimental Section). From the hysteresis behavior, the density of trapped charges (N_trap_) at the WS_2_/MoS_2_ and SiO_2_ interface is calculated using the following equation:

1 $${N}_{trap}=\frac{{C}_{oxide} * \Delta {V}_{TH}}{q}=\frac{{C}_{oxide} * |{V}_{TH}\left(forward\right)- {V}_{TH}(reverse)|}{q}$$

where C_oxide_ is the capacitance of the SiO_2_ back-gated oxide, V_TH_ is the threshold voltage obtained from the forward and reverse sweeps, and q is the quantity of electric charge. For a V_G_ swing of ± 30 V, the N_trap_ for the LHWM-synapse is obtained to be 5.44 × 10^12^ cm^−2^. Compared to the MoS_2_-based FET (Fig. S2), the negatively shifted V_TH_, higher SS, and larger trap density in the LHWM-FET are attributed to increased trap densities.

The opto-electronic characteristics of the LHWM-FET with the intrinsic photoresponsivity are investigated under the light illumination of 532 nm and 635 nm wavelength. Under light illumination, a negative shift in the V_TH_ and an increase of I_DS_ originate from the photo-generated carriers in the LHWM photoactive channel. After turning off the light illumination, the transfer curves of the LHWM-FET shifted in the negative direction under illumination of 532 nm and 635 nm wavelengths, and slowly returned to their initial state (i.e., dark condition) over time. In 635 nm light illumination, the initial dark state was reached in 360 s, and a long time of more than 780 s was required to return to the initial dark state in 532 nm light illumination. The negative shifted transfer characteristic, which is still observed when the lights are turned off, implies that recombination is suppressed even after the light was turned off by trapping the photo-generated carriers at the interface of the gate oxide and the semiconductor channel. In contrast, the transfer characteristics of the MoS_2_-FET returned to their initial state immediately after turning off the light illumination (Fig. S2). This is consistent with previous studies in which MoS_2_-FET responses to light illumination but quickly recovers to its initial state (i.e., volatile), making it difficult to implement long-term plasticity of the synaptic fuctions^20^. It indicates that the improved carrier trapping of the LHWM-FET is related to the WS_2_ layer. From these results, the presence of WS_2_ in the WS_2_/MoS_2_ lateral heterostructures supports that it may implement the photo-synaptic transistor with long-term plasticity by enhancing carrier trapping.

**Figure 3:**
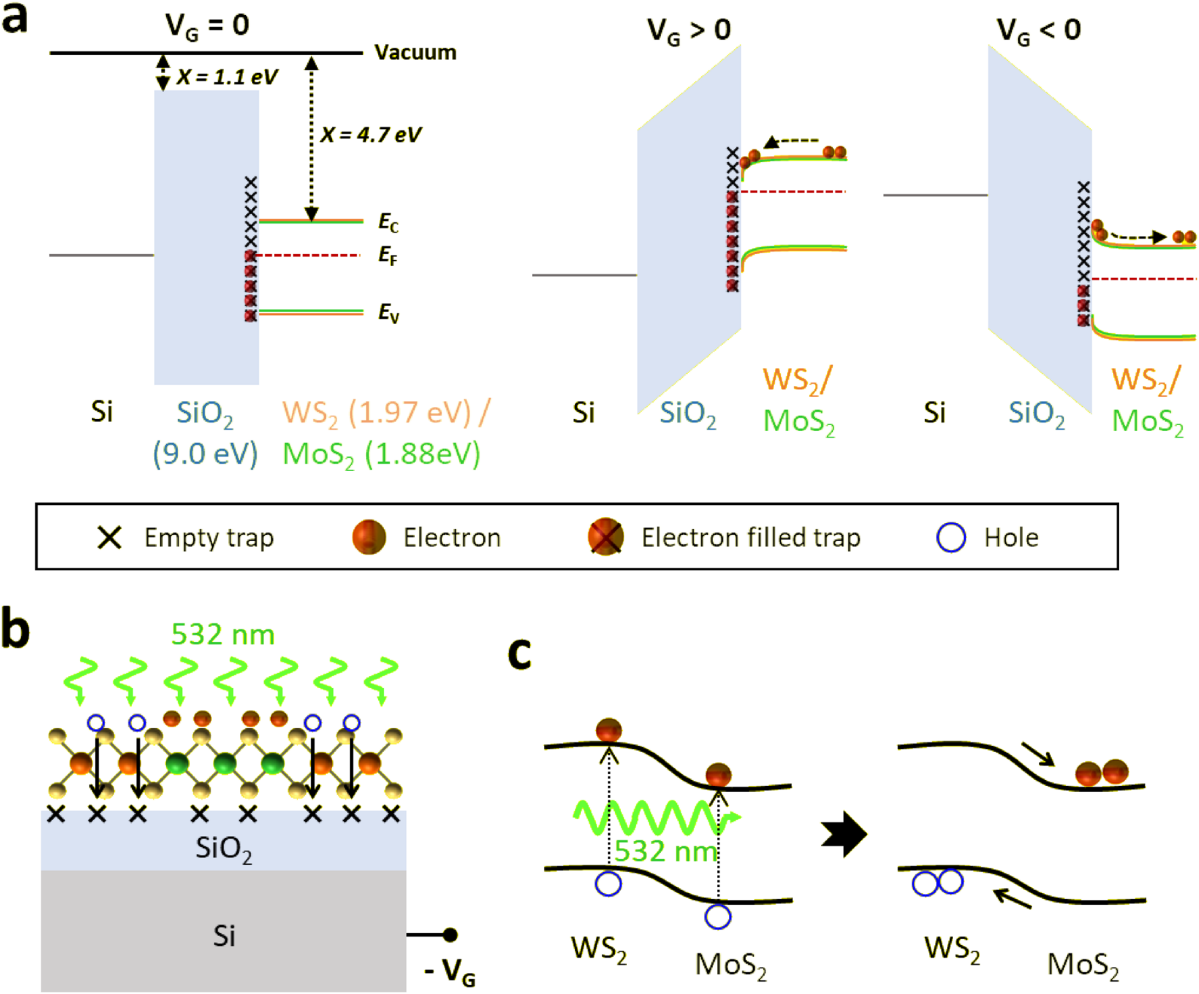
Energy band diagram and schematic illustration of WS_2_/MoS_2_-based photo-transistor for a synaptic device. (**a**) Energy band diagram of the WS_2_/MoS_2_ photo-transistor at different biases. (**b**) Schematic illustration of the WS_2_/MoS_2_ photo-transistor at the WS_2_/MoS_2_ and SiO_2_ interface with negative gate bias under 532 nm light illumination. (**c**) Band structures for the WS_2_ and MoS_2_ heterojunction under 532 nm light illumination.

To further understand the photo-synaptic behavior of the LHWM-FET, we investigated the mechanisms of charge trapping and de-trapping under dark and 532 nm light illumination conditions using time-resolved I_DS_ measurements (Figs. 3 and S3)^51–55^. The energy band diagram, including the bandgap energy (E_g_), the conduction band (E_c_), the valence band (E_v_), and the electron affinity (χ), was characterized using multi-dielectric energy band diagram software and PL responses, as supported by references^51–55^. The measurements were taken for 120 s at V_DS_ of + 1 V, V_G_ of + 30 V and − 30 V. Positive gate bias resulted in the gradual decrease of I_DS_ by trapping electrons from the WS_2_/MoS_2_ channel into the interface trap sites, leading to a decrease in the n-type characteristic (Figs. 3a and S3a,b). In contrast, the negative gate bias enhanced the n-type characteristic by releasing electrons from the interface trap sites into the WS_2_/MoS_2_ channel, resulting in a gradual increase of I_DS_ (Figs. 3a and S3c,d). Interestingly, the photo-generated holes were trapped at the interface by the negative gate bias, which in turn contribute to the conductance of the LHWM-FET, resulting in a significant increase in I_DS_ under light illumination (Figs. 3b and S3d).

**Figure 4:**
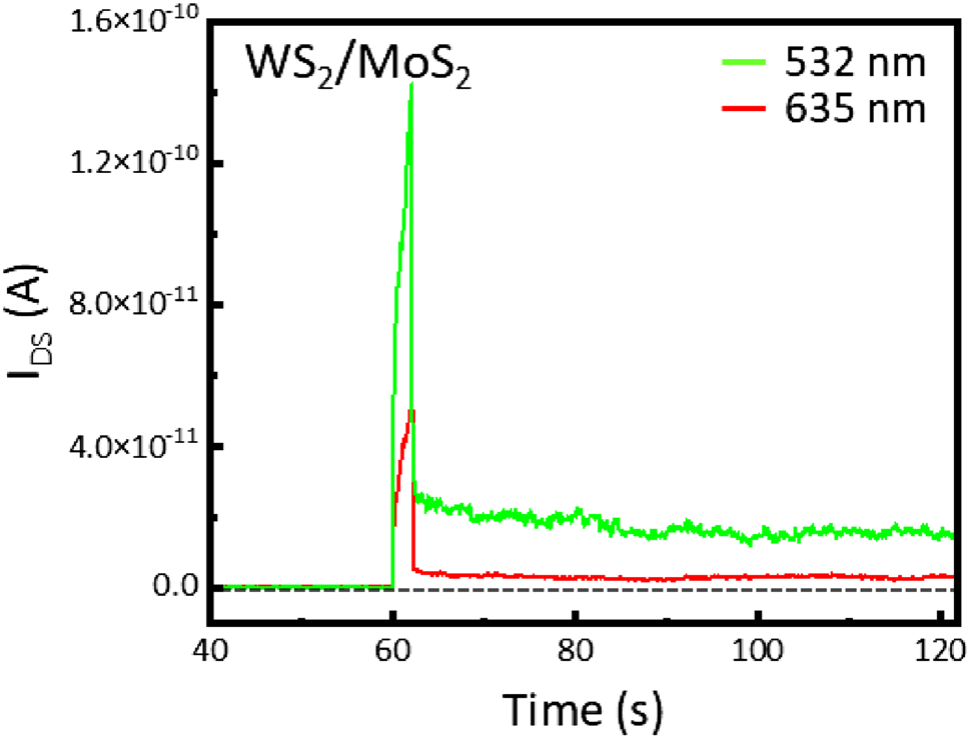
Photo-response behaviors of WS_2_/MoS_2_ heterostructure-based photo-transistor under illumination with different wavelength of 532 nm (green) and 635 nm (red). Source-drain currents (I_DS_) with respect to time of the photo-transistor illuminated by a single light pulse of 2 s at V_G_ =** − **30 V and V_DS_ = + 1 V. After a single light pulse of 532 nm and 635 nm, the current does not return to its initial state within 120 s.

To confirm the photo-synaptic behaviors, we investigated the conductivity variation of the LHWM-FET by applying a 2-s single pulse of 532 nm and 635 nm at V_G_ = − 30 V and V_DS_ = + 1 V (Fig. 4). For the MoS_2_-FET, the triggered photocurrents by a single light pulse rapidly returned to their initial sate as soon as the light pulse was turned off (Fig. S4). On the other hand, when applying the single light pulse, the LHWM-FET showed excitatory post-synaptic currents (EPSCs) that did not return to the initial state immediately. It is attributed that the type-II heterojunction of WS_2_ and MoS_2_ results in the photo-generated holes transferring to WS_2_, which enhances charge trapping generated at the WS_2_ and SiO_2_ interface (Fig. 3b,c). With the unique optical properties of the LHWM monolayer, the two different wavelengths may trigger different synaptic characteristics and thus exhibit the potential to implement multi-wavelength optoelectronic synapse.

**Figure 5:**
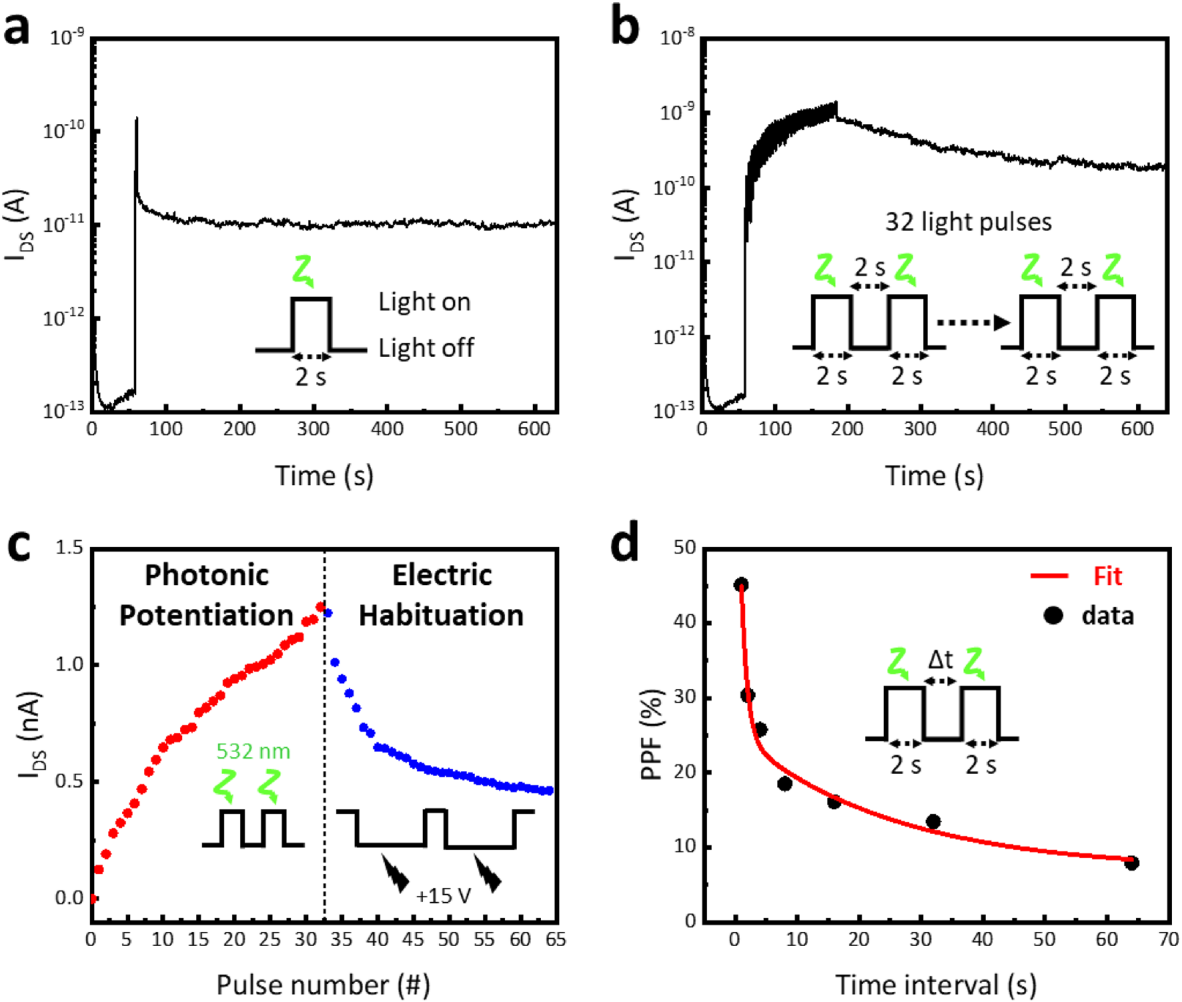
Characteristics of the LHWM-synapse at V_G_ =** − **30 V, V_DS_ = + 1 V, and a light pulse with a wavelength of 532 nm. (**a**) Excitatory post-synaptic current (EPSC) triggered by a single light pulse of 2 s. (**b**) EPSC triggered by 32 light pulses with an applied pulse width of 2 s and a time interval of 2 s. (**c**) The variation of I_DS_ versus the pulse trains. Potentiation and habituation processes were implemented by applying light pulses (photonic potentiation) and electrical pulses (electric habituation), respectively. (**d**) Paired-pulse facilitation (PPF) index [(A_2_–A_1_)/A_1_] as a function of pulse interval time (∆t) for the pulse width of 2 s.

To emulate the artificial synaptic characteristics of the LHWM-synapse, the effects of opto-electronic stimulation were investigated at the 532 nm light pulse under V_G_ of − 30 V and V_DS_ of + 1 V with 0.5 Hz repetition frequency (Fig. 5). First, the triggered EPSC of LHWM-synapse by an applied pre-synaptic light spike exhibited the retainable photocurrent for 570 s, representing that the trapped charge is remembered for the long term (Figs. 5a and S5a). Synaptic plasticity characteristics of the LHWM-synapse were further investigated by the rehearsal process. The analog multi-states of the long-term plasticity characteristic with linearity were observed by applying 32 consecutive light pulses with 0.5 Hz repetition frequency and 50% duty cycle (Fig. 5b,c). With 32 light pulse trains, the time-resolved I_DS_ is retainable due to the non-volatile charge trapping, indicating long-term potentiation behavior (Figs. 5b and S5b).

In contrast, for the MoS_2_-based synapse, the EPSC triggered by a single pulse not only returned quickly to the initial state, but also exhibited short-term plasticity characteristics with nonlinearity in 32 consecutive light pulses (Fig. S5c,d). The LHWM-synapse was potentiated by applying 32 consecutive light pulses of 532 nm wavelength at a constant V_G_ of − 30 V and V_DS_ of + 1 V with a 0.5 Hz repetition frequency (Fig. 5c). The analog multi-states which implement LTP characteristics in the synaptic device require linear behavior to ensure high recognition accuracy. With 32 light pulse trains, the photonic potentiation process shows an increase of the EPSC gradually from 125 pA to 1.25 nA, and the 32 analog states have good linearity. In 32 analog multi-states, the EPSC ratio triggered by the 1st pulse and 32nd pulse is 9.97. The non-linearity factor of LHWM-synapse is extracted from the potentiation behavior:

2 $${I}_{LTP}={I}_{min}+{I}_{o}*(1-{e}^{-vx})$$

where ${I}_{min}$ is minimum current, $x$ is the number of pulse, ${I}_{o}$ is the fitting parameter, and $v$ is the non-linearity factor. The non-linearity factor of LTP behavior for the LHWM-synapse is obtained to be 0.06. In the LTP behavior of LHWM-synapse, the good linearity of 32 conduction states may implement 5-bit memory with high recognition accuracy by the photonic potentiation. The response to the stimulus in the LHWM-synapse is characterized by a depression effect, induced by applying 32 electrical pulses with a V_G_ amplitude of + 15 V and a constant V_DS_ of + 1 V. Each pulse had a duration of 8 s, interspersed with intervals of 2 s, under dark conditions. Electrical stimulus implemented gradual long-term depression (LTD) behavior of the LHWM-synapse. The non-linearity factor of LHWM-synapse is extracted from the depression behavior:

3 $${I}_{LTD}={I}_{max}-{I}_{o}*(1-{e}^{-v(x-32)})$$

where ${I}_{max}$ is maximum current, $x$ is the number of pulse, ${I}_{o}$ is the fitting parameter, and $v$ is the non-linearity factor. The non-linearity factor of LTD behavior for the LHWM-synapse is obtained to be 0.18. With the electrical stimulus, the LHWM-synapse implemented 32-LTD behavior for 5-bit memory.

The paired-pulse facilitation (PPF) characteristic of the LHWM-synapse (Fig. 5d), where the second pre-synaptic spike caused a larger post-synaptic current than the first pre-synaptic spike, was investigated at the light pulse width of 2 s:

4 $$PPF= \frac{{A}_{2}-{A}_{1}}{{A}_{1}}$$

where ${A}_{1}$ and ${A}_{2}$ are the amplitudes of the first EPSC and second EPSC, respectively. The maximum PPF index of the LHWM-synapse was 45.2% for a 1 s time interval between two consecutive light pulses. As the $\Delta t$ time interval increased from 1 to 64 s, the PPF index gradually decreased from 45.2% to 7.9%. The relationship between $\Delta t$ and PPF index was fitted by the double-exponential function:

5 $$PPF=1 + {A}_{1}*exp\left(-\Delta t/{\tau }_{1}\right) + {A}_{2}*exp(-\Delta t/{\tau }_{2})$$

where ${A}_{1}$ and ${A}_{2}$ are the initial facilitation magnitudes, and ${\tau }_{1}$ and ${\tau }_{2}$ are the characteristic relaxation times. For the pulse width of 2 s, the derived relaxation times were found to be ${\tau }_{1}$ = 0.97 s and ${\tau }_{2}$ = 25.3 s, which are consistent with the response of a biological synapse.

## Conclusion

In conclusion, we have identified the potential of heteronanostructures of WS_2_/MoS_2_ monolayer as promising candidates for artificial photo-synaptic devices with long-term plasticity and 5-bit memory. By utilizing the inherently high photoresponsivity and enhanced charge trapping capability of these structures, we successfully emulated photo-synaptic behaviors. To investigate the applicability of the LHWM-synapse, we applied both optical and electrical stimuli, and examined various synaptic properties such as the retention of excitatory post-synaptic currents, potentiation, habituation, nonlinearity factor, and paired-pulse facilitation. These results suggest that lateral heterojunctions in 2D material-based synapse could provide a simple and scalable nanomanufacturing method for achieving analog multi-states with long-term plasticity and high learning accuracy. While further research is required to refine synaptic weights by reducing pulse width and applied voltage, and to explore different wavelengths for the practical implementation of versatile photo-synapses, our findings indicate the potential of 2D material-based photo-synapses as a pioneering approach. These devices are at the forefront of not only enabling next-generation neuromorphic and optoelectronic computing systems but also expanding their reach into fields such as intelligent sensing, advanced human–machine interfaces, precise biomedical diagnostics, and innovative wearables.

## Experimental section

*Materials* Mo(CO)_6_ (≥ 99.9%) and W(CO)_6_ (≥ 99.99%) were purchased from Sigma-Aldrich Co., USA. H_2_S (99.9%) gas was purchased from Noblegas Co., Republic of Korea. H_2_ (99.999%) and Ar (99.999%) gases were purchased from Samogas Co., Republic of Korea. Polymethyl methacrylate (PMMA) was purchased from MicroChem Co., USA. Methyl isobutyl ketone (MIBK) was purchased from Kayaku Co., Japan. Isopropyl alcohol (IPA) and acetone solvents were purchased from Duksan Pure Chemicals Co., Republic of Korea. A p-type Si wafer with a 300-nm thick SiO_2_ layer (SiO_2_/Si, < 0.005 Ω cm) was purchased from iTASCO Co., Republic of Korea.

*Growth of laterally-heterostructured WS*_*2*_*/MoS*_*2*_* monolayer* The laterally-heterostructured WS_2_/MoS_2_ (LHWM) monolayer was grown on a SiO_2_/Si wafer using a metal–organic chemical vapor deposition (MOCVD) system with a shower-head-type reactor. The substrate was loaded into a MOCVD chamber at a temperature of 400 °C. A large amount of H_2_ (600 sccm) and H_2_S (600 sccm) gases were injected into the process chamber, and the working pressure was controlled to be 40 Torr to facilitate the 2D lateral growth of the WS_2_/MoS_2_ heterostructure. To regulate the flow rate of Mo(CO)_6_ and W(CO)_6_ precursors, a circulation chiller system (set at 2 °C) connected to precursor canisters was used with a carrier gas of 0.3 sccm Ar. Firstly, the MoS_2_ monolayer was grown under a working pressure of 40 Torr and a temperature of 400 °C for 2 h. A two-step process of simple sequential MOCVD was used to form the LHWM monolayer. After the growth of the MoS_2_ monolayer, the flow of the Mo(CO)_6_ precursor was terminated, and the injection of H_2_S and H_2_ gases into the working chamber was maintained for 1 h at a pressure of 40 Torr. This stabilization process, which involved purging precursor and byproducts, was carried out for 1 h. Then, the second W(CO)_6_ precursor was injected to grow WS_2_ thin films. The LHWM monolayer was formed by the lateral growth of WS_2_ at the edge of the MoS_2_ monolayer.

*Fabrication of the field-effect transistor (FET) for a photo-synaptic device* A back-gated FET based on the LHWM monolayer was fabricated using the e-beam lithography method. First, the PMMA 950 C4 e-beam resist was spin-coated on the WS_2_/MoS_2_ monolayer at 500 rpm for 5 s and 5000 rpm for 40 s. The spin-coated sample was baked at 180 °C for 90 s. The ER layer was patterned with an area dose of 300 µC cm^−2^ at 30 keV exposure using a field-emission scanning electron microscopy (SEM) system (FEI Sirion 400, Czech). The LHWM-based FET device has a line width of 2 µm and a spacing of 300 nm. Then, the e-beam exposed sample was developed in a mixture solution of MIBK/IPA (1:3 volume concentration ratio) for 30 s. A 100 nm Au/10 nm Ti film was deposited for the source and drain electrodes at a working pressure of ~ 10^−8^ Torr and a deposition rate of ~ 0.2 Å/s by an e-beam evaporator system (IVT, Republic of Korea). To peel off the metal film on the ER layer, a lift-off process was carried out with an acetone solvent for 24 h. Then, the sample was rinsed in fresh acetone and IPA, and was blow-dried with N_2_ gas. As a result, an LHWM-back-gated FET was prepared for use a photo-synaptic transistor.

*Characterization* The structural and morphological properties were characterized by field-emission SEM at accelerating voltages of 10 kV (Hitachi S-4800, Japan) and 1 kV (Zeiss Sigma 300 VP, Germany). Raman and photoluminescence (PL) were measured using a confocal Raman spectroscopy (Renishaw inVia, UK) system with a 488 nm laser of 100 µW power. The electrical properties of the FETs were characterized using a measurement system consisting of a semiconductor parameter analyzer (Keysight HP 4156A, USA) with a pulse generator (Keysight HP 41501, USA) and a probe station (Cascade Microtech Alessi REL-5500, USA). In electrical characterization, the on/off current ratio is calculated as the ratio of maximum I_DS_ to minimum I_DS_. The threshold voltage (V_TH_) is estimated as the linear region extrapolation of the slope of I_DS_^0.5^ versus V_G_ curve, and the sub-threshold swing (SS) is defined as the inverse of the slope of the log I_DS_ versus V_G_ curve.

### Supplementary Information

Supplementary Figures.

## Data Availability

All data generated or analysed during this study are included in this published article (and its Supplementary Information files).
