# Supplementary material for: Lateral heterostructures of WS2 and MoS2 monolayers for photo-synaptic transistor
Source: Sci Rep. 2024 Mar 22;14:6922. doi: 10.1038/s41598-024-57642-6 (PMC10959970; doi:10.1038/s41598-024-57642-6)
Supplement: Supplementary file 1 — Supplementary Figures. [file 41598_2024_57642_MOESM1_ESM.docx]

Supporting Information

Lateral Heterostructures of WS_2_ and MoS_2_ Monolayers for Photo-Synaptic Transistor

Jaeseo Park^1,^**^†^**, Jun Oh Kim^1,^**^†^**, and Sang-Woo Kang^1,2,^*

*^1^Strategic Technology Research Institute, Korea Research Institute of Standards and Science, Daejeon 34113, Republic of Korea*

*^2^Precision Measurement, University of Science and Technology, Daejeon 34113, Republic of Korea*

**^†^**Jaeseo Park and Jun Oh Kim contributed equally to this work

*Corresponding author. E-mail: swkang@kriss.re.kr


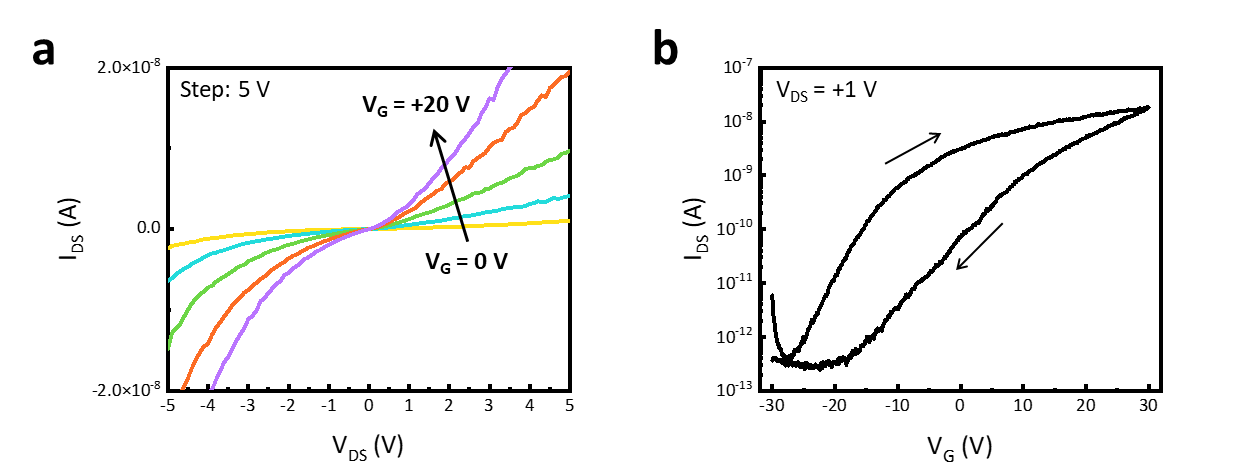


Figure S1. Output and transfer characteristics of the laterally-heterostructured WS_2_/MoS_2_-FET. (a) Output curves of I_DS_-V_DS_ for various V_G_ values from 0 V to +20 V in step of 5 V. (b) Transfer curve of I_DS_-V_G_ at V_DS_ of +1 V with a sweeping range from −30 V to +30 V.


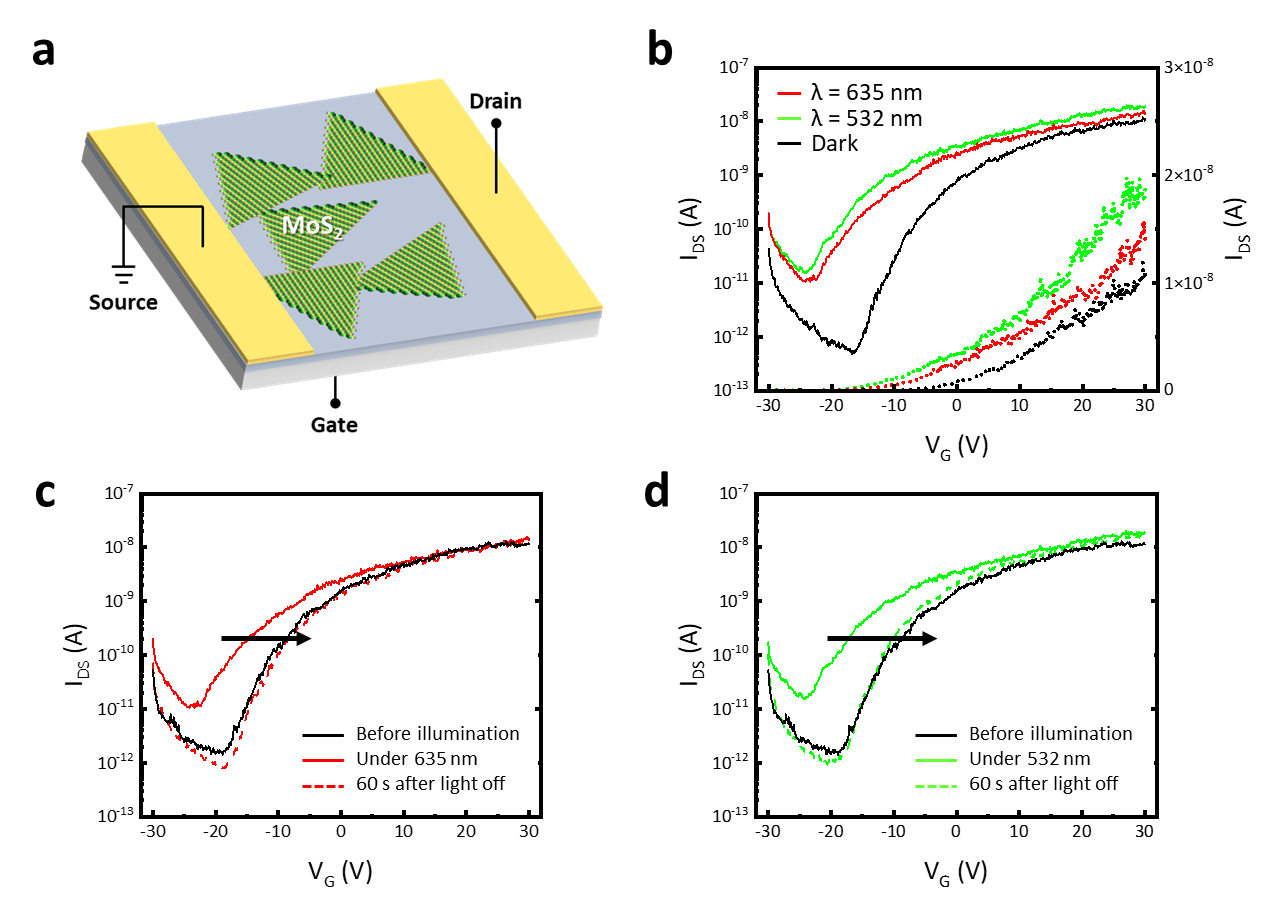


Figure S2. Device structure and transfer characteristics of the MoS_2_-based back-gated FET. (a) Schematic illustration of the back-gated MoS_2_-based photo-transistor. Transfer characteristics with a wavelength of (b) 635 nm and (c) 532 nm are shown. Based on the I_DS_-V_G_ transfer characteristic of the MoS_2_-FET, the on/off ratio of ~10^4^, threshold voltage (V_TH_) of −16.98 V, and sub-threshold voltage (SS) of 3.93 V/dec are calculated (Experimental Section).


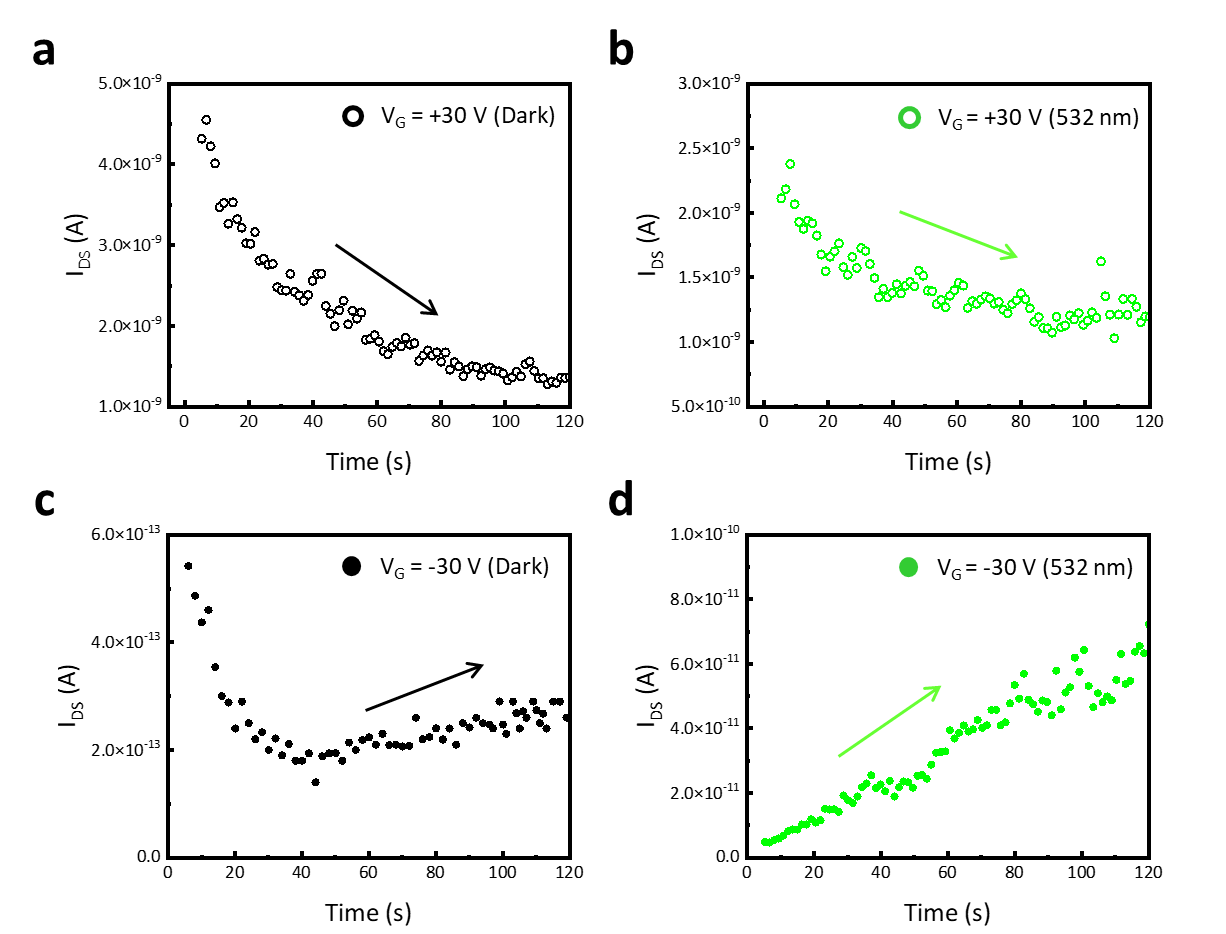


Figure S3. Time-resolved I_DS_ under dark and 532 nm light illumination conditions at V_DS_ of +1 V for 120 seconds. At V_G_ of +30 V, the time-resolved I_DS_ under (a) dark and (b) 532 nm light illumination conditions are shown. At V_G_ of −30 V, the time-resolved I_DS_ under (c) dark and (d) 532 nm light illumination conditions are shown.


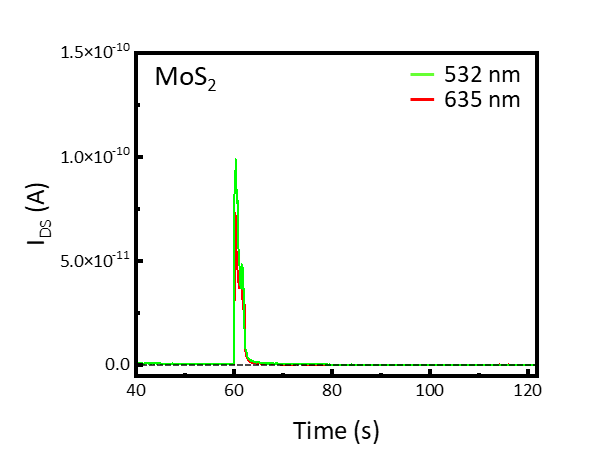


Figure S4. Photo-response behavior of the MoS_2_-based photo-transistor under the illumination with different wavelengths of 532 nm (green) and 635 nm (red). The I_DS_ of the photo-transistor was measured to time when illuminated by a single light pulse of 2 seconds at V_G_ = −30 V and V_DS_ = +1 V. After a single light pulse of 532 nm and 635 nm, the current immediately returned to its initial state.


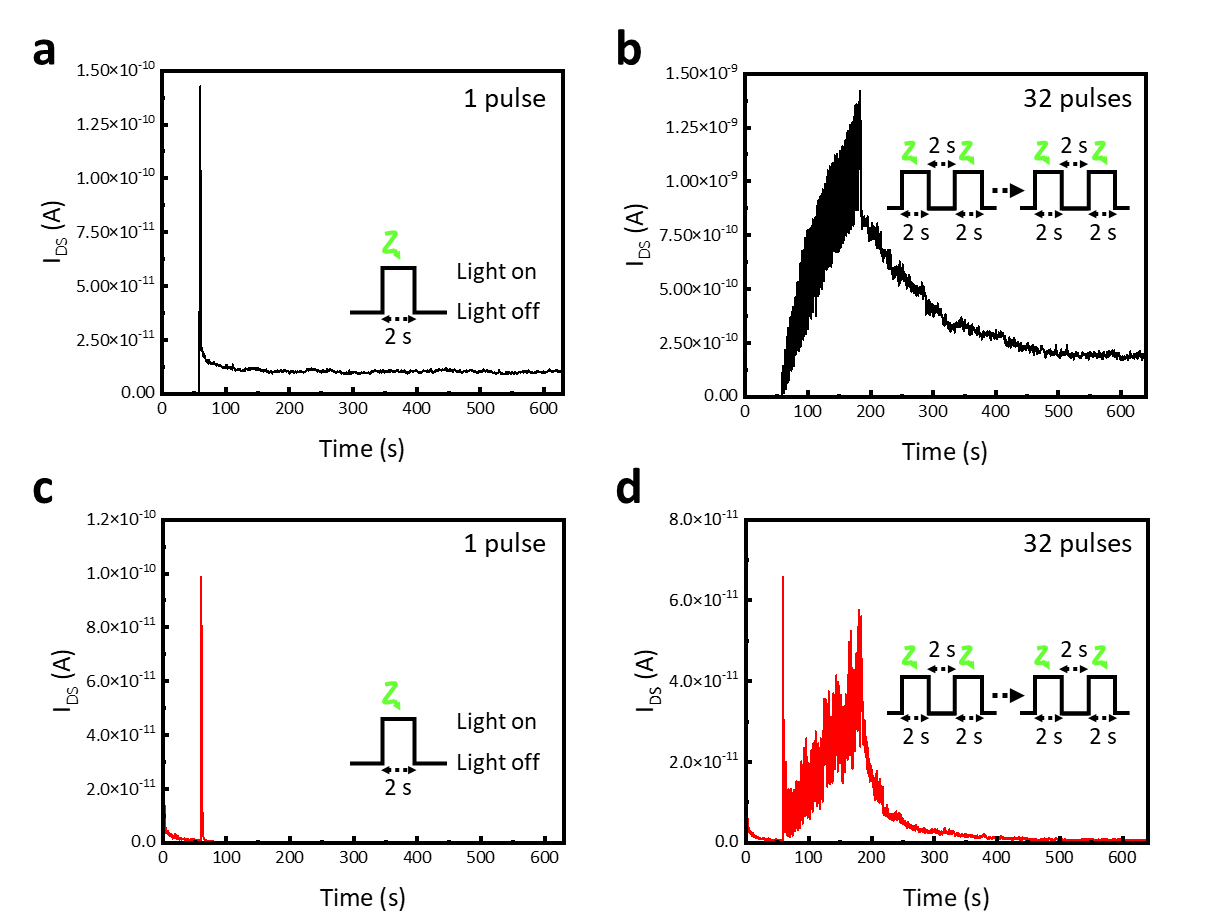


Figure S5. Characteristics of (a-b) WS_2_/MoS_2_- and (c-d) MoS_2_-based photo-synaptic transistor under V_G_ = −30 V, V_DS_ = +1 V, and a 2-second light pulse with a wavelength of 532 nm. (a,c) EPSC triggered by a single light pulse. (b,d) EPSC triggered by 32 light pulses with an applied pulse width and time interval 2 seconds.
